# Adaptive dose-finding designs to identify multiple doses that achieve multiple response targets

**DOI:** 10.1186/1745-6215-14-S1-O78

**Published:** 2013-11-29

**Authors:** Simon Bond, Adrian Mander, John Todd, Linda Wicker, Frank Waldron-Lynch

**Affiliations:** 1 Cambridge Clinical Trials Unit, Cambridge University Hospitals NHS Foundation Trust, Cambridge, UK; 2 MRC Biostatistics Unit Hub for Trials Methodology Research, Cambridge, UK; 3 JDRF/Wellcome Trust Diabetes and Inflammation Laboratory, Cambridge Institute for Medical Research, Cambridge, UK

## 

The objective of the DILT1D trial is to identify doses of interleukin-2 that achieve targeted increases in the T regulatory cell population in recently diagnosed type 1 diabetes. DILT1D aims to accurately identify a minimally effective dose and a maximal dose in a limited number of patients (40) that may be repeatedly administered in later phase trials.

The dose is administered subcutaneously so can be chosen from a continuous range up to a limit chosen on tolerability grounds. The design has an initial learning phase where pairs of patients are assigned to five pre-assigned doses. The next phase is fully sequential with an interim analysis after each patient to determine the choice of dose based on the optimality criterion to minimise the determinant of the covariance of the estimated target doses. The dose-choice algorithm assumes that a specific parametric dose-response model is the true relationship, and so a variety of models are considered at the interim and human judgement involved in the overall decision.

Simulation studies found that having two targets and assuming a model with a small number of parameters (≤3) leads to a choice of doses that are not near to the target doses. Fitting models with greater flexibility with more parameters (≥4) results in a choice of doses near to the target doses. Overall the design is efficient and seamlessly combines the initial learning and subsequent confirmatory stages.

